# The effect of avatar identity on spontaneous perspective-taking in patients with schizophrenia

**DOI:** 10.1038/s41537-024-00551-4

**Published:** 2025-01-25

**Authors:** Pei Xie, ChaoZheng Huang, XiaoQuan Wang, HanBin Sang, AiBao Zhou

**Affiliations:** 1 School of Psychology, Sichuan Normal University, Chengdu, China; 2 School of Psychology, Northwest Normal University, Lanzhou, China; 3 School of Judicial Police, Gansu University of Political Science and Law, Lanzhou, China; 4 Tianshui Third People’s Hospital, Tian Shui, China; 5 School of Teacher Education, Qiongtai Normal University, Haikou, China; 6 Key Laboratory of Child Cognition & Behavior Development of Hainan Province, Haikou, China

**Keywords:** Human behaviour, Schizophrenia

## Abstract

Controversy exists regarding whether the spontaneity of altercentric intrusion is impaired in patients with schizophrenia during implicit visual perspective-taking tasks. This study explored the characteristics of spontaneous visual perspective-taking in patients with schizophrenia and the effect of an avatar identity on their perspective-taking. We recruited 65 patients with schizophrenia and 65 healthy participants to complete 4 visual perspective-taking experiments for uncued other-avatar and self-avatar tasks and cued other-avatar and self-avatar tasks. In uncued other-avatar experiments, healthy controls showed a significant reduction in accuracy and an increase in response latency when the number of visible discs differed from that seen by the other-avatar (inconsistent condition), indicating altercentric intrusion. However, patients with schizophrenia did not exhibit this effect. In uncued self-avatar experiments, when the avatar was defined as the participant themselves, patients with schizophrenia did not show spontaneous perspective-taking. However, in cued other-avatar experiments, they showed altercentric intrusion in response latency, and in cued self-avatar experiments, they showed altercentric intrusion in accuracy and response latency. These results suggest that patients with schizophrenia have the tendency to spontaneously adopt the perspective of others, which is predicated on their awareness of the existence of perspectives. In addition, the avatar’s identity as a stranger hinders the spontaneous perspective-taking processes in patients with schizophrenia.

## Introduction

Patients with schizophrenia (SZ) face significant challenges in social interactions because of their impaired social cognitive abilities^1,2^. They find it difficult to infer others’ mental states and understand the differences between others’ and their own thoughts, beliefs, intentions, and feelings^3–5^. This is the ability of theory of mind (ToM)^6,7^. A previous study revealed that healthy individuals possess the capacity to track others’ mental states in a relatively spontaneous, rapid, and efficient manner, a capability referred to as “implicit ToM”^8^. Although numerous studies have suggested that ToM is typically impaired in patients with SZ^9,10^, no consensus currently exists on the manifestations of implicit processes that underpin ToM. It is necessary to clarify the psychological mechanisms underlying this impairment through further empirical research, to provide assistance for interventional treatment and improve patients’ social cognitive abilities.

Researchers generally demonstrate individuals’ capacity of ToM by investigating visual perspective-taking, which recognizes the distinction between one’s own and others’ perspectives and allows individuals to restrain their perspective when tracking or considering that of others^11–13^. By engaging in visual perspective-taking, individuals perceive the visual experiences of others and anticipate and interpret their behaviors, which facilitates social interactions and enables them to adapt to social environments^14,15^. Implicit visual perspective-taking primarily involves the automatic and unconscious process of adopting others’ perspectives^16^. It can overcome the influence of other cognitive impairments associated with SZ and reliably represent patients’ ToM dysfunction^17^. However, studies have reported conflicting results^4,18,19^.

Simonsen et al.^18^ adopted the dot perspective task proposed by Samson et al.^13^ to examine visual perspective-taking performance in SZ. The experimental material comprised a picture of a human avatar placed in the middle of a room. The human avatar alternated between facing the left and right walls of the room, with both walls having several discs. In half of the pictures, the number of discs visible to the participants is consistent with that seen by the human avatar, whereas in the other half, it is inconsistent. During the experiment, the participants were asked to judge the number of discs from either their own perspective or that of the avatar. When the number of discs visible to the participants differed from that seen by the avatar, and if their judgments deteriorated compared with the consistent condition, it indicated the spontaneous adoption of the other’s perspective (altercentric intrusion). Simonsen et al.^18^ observed compared to controls, increased altercentric intrusion in patients with schizophrenia.

However, another study found reduced altercentric interference in patients with SZ. In task of Kronbichler et al.^4^, participants were only required to determine whether the number of discs on the wall is consistent with the given number, and there is no clue about the perspective during the experiment. The authors believe that this design can minimize the perspective-taking effects caused by cues. Their study indicated patients with SZ are less likely to spontaneously compute the visual perspectives of others. These conflicting outcomes may stem from task design variance. The relevant debate centers on which design (cued or uncued) accurately reflects patients’ implicit visual perspective-taking.

In patients with SZ, spontaneous processing is crucial for demonstrating implicit visual perspective-taking ability. O’Grady et al.^20^ suggested that perspective-taking may be ineffective because of the lack of awareness of its potential relevance in uncued tasks; drawing attention to an avatar triggers a spontaneous visual perspective-taking process. In a task proposed by Kronbichler et al.^4^, participants did not receive any instructions about perspectives. One of the clinical impairments of patients with SZ is self-disorders, that manifest as alterations in the organization or sensation of one’s field of awareness, impairing their ability to recognize the existence or meaning of objects accurately^21^. This indicates patients with SZ, who experience difficulties in attending to the external world, may struggle with being aware of the existence of perspective-taking, especially when the avatar is presented as an unfamiliar stranger. To examine the ability of implicit visual perspective-taking in patients with SZ, we redefined the identity of human avatars to increase their likelihood of being noticed so as to clarify the ability of patients’ ToM.

In studies employing the dot perspective task to assess visual perspective-taking abilities, human avatars are often characterized as strangers to assess whether participants can adopt others’ perspectives^13,22^. However, in an implicit visual perspective-taking task, the focus shifts to spontaneous processes^17^. Consequently, defining the human avatar as the participant themselves enhances their attention toward the avatar and enables the exploration of their spontaneous abilities. When participants realize that the human avatar represents them, this close relationship with the self potentially prompts them to become aware of the existence of visual perspective-taking.

We conducted a cued dot perspective task to ensure the acceptability of our study. If the self-as-avatar uncued task validated that patients with SZ were unable to spontaneously adopt the avatar’s perspective, it would indicate an impairment of their spontaneous processes. However, the information on perspective-taking provided for the uncued tasks could also be too limited to allow participants to connect perspective-taking with the current task. According to O’Grady et al.^20^, the rapid, unconscious, and involuntary computation of others’ perspectives necessitates that participants direct their attentional system toward perspective-taking. Therefore, we independently investigated the perspective-taking of patients with SZ toward other avatars and self-avatars in the cued dot-probe tasks to verify their spontaneous ability to adopt the perspectives of others.

In summary, our research elucidates the performance of implicit ToM in patients with SZ by comparing their spontaneous perspective-taking of other- and self-avatars in uncued and cued dot-probe tasks. The self-avatars in the current study enabled the separation of the measured content’s spontaneous perspective-taking from the self–other distinction process. This revealed the psychological mechanism underlying this process by clarifying the patients’ abnormal spontaneous adoption of others’ perspectives.

## Experiment 1a: uncued task of others’ perspective-taking in patients with schizophrenia

### Methods

#### Participants

An a priori power analysis was conducted using G*Power version 3.1.9.2^23^ to determine the minimum sample size required to test the study hypothesis. Results indicated the required sample size to achieve 80% power for detecting a medium effect (*f* = 0.2), at a significance criterion of *α* = 0.05, was *N* = 42 for 2 × 2 repeated measures in the analysis and interpretation of ANOVA. To avoid insufficient sample size due to participants abandoning the experiment, a total of 32 patients with SZ and 30 healthy controls were recruited for Experiments 1a and 1b. All patients with SZ were recruited from the Tianshui Psychiatric Hospital, Gansu Province, China. The patients fulfilled the diagnostic criteria provided in the tenth revision of the International Classification of Diseases-10 (ICD-10) and were diagnosed by two experienced attending doctors. The exclusion criteria were substance abuse, history of severe brain trauma, and other physical diseases. All patients received antipsychotic medication (mean chlorpromazine equivalent = 257.55 mg) during the experiment (Table 1).

**Table 1:** Basic characteristics of participants.

	Schizophrenia (*n* = 32) (*M* ± SD)	Healthy controls (*n* = 30) (*M* ± SD)
Age (y)	30.22 ± 7.63	29.57 ± 9.78
Years of education	9.81 ± 3.12	13.73 ± 2.83
Gender (male/female)	19/13	18/12
Duration of illness (y)	7.93 ± 4.85	
SAPS	9.07 ± 9.59	
SANS	13.38 ± 10.95	

*SAPS* Scale for Assessment of Positive Symptoms, *SANS* Scale for Assessment of Negative Symptoms.

The healthy controls and patients were matched for sex, age, educational level, and residence. The exclusion criteria for the healthy controls were substance abuse, smoking, drinking problems, history of psychotic disorders, history of severe brain trauma, and other physical diseases. The healthy controls who completed the entire procedure received 30 RMB as compensation for their participation. The study was approved by the Ethics Boards of Northwest Normal University. Informed consent was obtained from all participants. The research was performed completely in accordance with the relevant guidelines and regulations and the Declaration of Helsinki.

#### Stimuli and design

We used the dot perspective task adapted by Kronbichler et al.^4^ from ref.^13^. The stimulus pictures presented a side view of a room, with the left, middle, and right walls clearly visible. Red discs appeared on the walls positioned on the left or right sides, either individually on one wall or simultaneously on both. At the center of the room was a human avatar (male or female). Two conditions were considered: one where the avatar faced left and the other where it faced right. The key difference was in the number of red discs visible to the participants and the avatar. In one condition, both could see an equal number of red discs, whereas in the other, the number of visible red discs differed. The number of red discs on the walls ranged from 0 to 3. We adopted a 2 (group: patients with SZ and healthy controls) × 2 (consistency: consistent and inconsistent) factorial design.

#### Uncued visual perspective-taking task

**Fig. 1:**
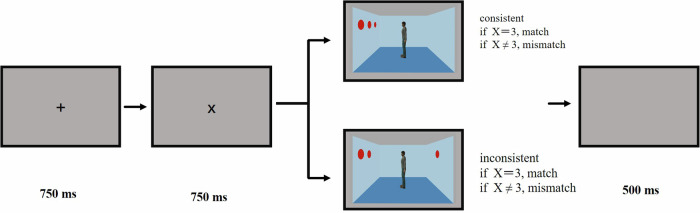
An illustration of the trial procedure in the uncued visual perspective-taking task.

The participants were invited to the laboratory for the task, which was programmed using E-Prime 2.0. They were seated 60 cm from the monitor screen, with their head position fixed using a chin rest. Each trial began with the presentation of a black central fixation cross “+” for 750 ms, followed by the presentation of one number from 0 to 3 for 750 ms, which cued the participant to verify the number of red discs in the next image. Subsequently, an image of the room was presented, and the trial continued until the participant pressed a key, which led to a 500 ms blank screen before proceeding to the next trial. The participant was required to press a key (F or J) to confirm whether the number of discs in the room matched the previously seen number. Key presses were balanced among the participants (Fig. 1). The experimental instructions informed the participants that the stranger standing in the middle of the room was irrelevant, requiring participants to identify whether the number of red discs in the room matched the previously seen number, without any reference to perspectives.

According to the experimental design, in the condition where the participant’s perspective matched the avatar’s, there were 24 trials each in which the answers were “yes” and “no.” Similarly, when the participant’s perspective did not match that of the avatar, there were 24 trials for each answer (“yes” and “no”). In total, 96 trials were presented for all stimuli. To prevent the same conditions from occurring three consecutive times, all trials were presented pseudo-randomly during the experiment. Additionally, 16 filler trials were included, in which the cue number was 0 and there were no red discs in the image. Including the 12 practice trials, the total number of trials was 124. At the beginning of the practice phase, the assistant patiently explained the experimental content to the participants until they had fully understood it. The formal experiment lasted ~8–10 min.

In some trials in which the correct answer was “no,” the number of red discs did not correspond to either the participant’s perspective or the avatar’s, making these trials particularly easy to process and potentially confounding the results. Therefore, during the final analysis, we only analyzed conditions in which the correct answer was “yes.”

#### Statistical analysis

We excluded the data from the practice trials. The response latency results were preprocessed, and trials with errors were removed. Only the response latencies falling within ± 3 standard deviations of each participant’s average response latency were considered. Ten patients (eight with accuracy rates not exceeding 80% and two with an average response latency exceeding 3000 ms) and six healthy controls (with accuracy rates not exceeding 80%) were excluded from the analysis. All data were analyzed using IBM® SPSS® Statistics Version 23.0 via a 2 (group: patients with SZ and healthy controls) × 2 (consistency: consistent and inconsistent) repeated measures ANOVA.

### Results

#### Accuracy rate

**Fig. 2:**
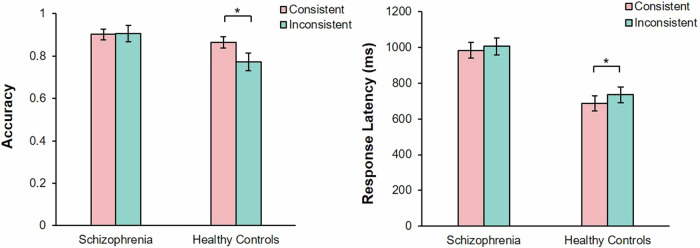
Shows the comparison of accuracy (left) and response latency (right) between consistent perspective and inconsistent perspective in patients with schizophrenia and healthy controls under the uncued stranger avatar task. The error bars represent 1 standard error. *P < 0.05.

A significant main effect of the group was observed, *F* (1, 60) = 4.23, *P* < 0.05, partial *η*^2^ = 0.07. There was no significant main effects of consistency, *F* (1, 60) = 3.81, *P* = 0.06. There was a significant interaction between group and consistency, *F* (1, 60) = 4.04, *P* < 0.05, partial *η*^2^ = 0.07. The simple effect analysis showed that, healthy controls showed higher accuracy rate in consistent perspectives condition (*M* = 0.86, 95% CI [0.81, 0.92]) than inconsistent perspectives condition (*M* = 0.77, 95% CI [0.69, 0.85], *t* (29) = 1.97, *P* < 0.05, Cohen’s *d*_*z*_ = 0.36. Patients with SZ showed no significant difference in accuracy rates between consistent perspectives (*M* = 0.91, 95% CI [0.83, 0.98]) and inconsistent perspectives (*M* = 0.90, 95%CI [0.85, 0.95], *t* (31) = 0.20, *P* = 0.84) (Fig. 2).

#### Response latency

There were significant main effects of group, *F* (1, 44) = 20.56, *P* < 0.001, partial *η*^2^ = 0.32, and consistency, *F* (1, 44) = 14.03, *P* < 0.01, partial *η*^2^ = 0.24. There was no significant interaction between group and consistency, *F* (1, 44) = 1.78, *P* = 0.19. The simple effect analysis showed that, healthy controls showed longer response latency in inconsistent perspectives condition (*M* = 734.10, 95% CI [643.19, 825.01]) than in consistent perspectives condition (*M* = 685.62, 95% CI [600.05, 643.188], *t* (23) = 3.45, *P* < 0.05, Cohen’s *d*_*z*_ = 0.70). Patients with SZ showed no significant difference in response latency between consistent perspectives (*M* = 1005.29, 95% CI [910.34, 1100.25]) and inconsistent perspectives (*M* = 982.30, 95% CI [892.92, 1071.67], *t* (21) = 1.81, *P* = 0.85) (Fig. 2).

### Discussion

In Experiment 1a, we adopted an uncued task to explore the implicit visual perspective-taking ability of patients with SZ. The accuracy and response latency results indicated that when the avatar’s perspective was inconsistent with that of the participants, the judgments of healthy controls were affected, whereas those of the patients were not. This finding is consistent with research conducted by Kronbichler et al.^4^, which suggested that patients with SZ do not display altercentric intrusion and may not adopt others’ perspectives spontaneously. To verify the influence of avatar salience on the spontaneity of patients with SZ in Experiment 1b, we replaced the avatar’s identity with that of the participants.

## Experiment 1b: uncued task of self-perspective-taking in patients with schizophrenia

### Methods

#### Stimuli, design, and procedure

We used the same stimuli and procedures as in Experiment 1a, except for the avatar identity (Fig. 1). The experimental instructions informed participants that the avatar in the room represented themselves and was not directly involved in the task; they were required to count whether the number of red discs in the room matched the previously seen number. The instructions did not include directives related to perspectives. The experiment used a 2 (group: patients with SZ and healthy controls) × 2 (consistency: consistent and inconsistent) factorial design.

#### Statistical analysis

The data were excluded based on the same method as in Experiment 1a. Eight patients (five with accuracy rates not exceeding 80% and three with an average response latency exceeding 3000 ms) and four healthy controls (with accuracy rates not exceeding 80%) were excluded from the analysis. All data were analyzed using IBM® SPSS® Statistics Version 23.0 via a 2 (group: patients with SZ and healthy controls) × 2 (consistency: consistent and inconsistent) repeated measures ANOVA.

### Results

#### Accuracy rate

**Fig. 3:**
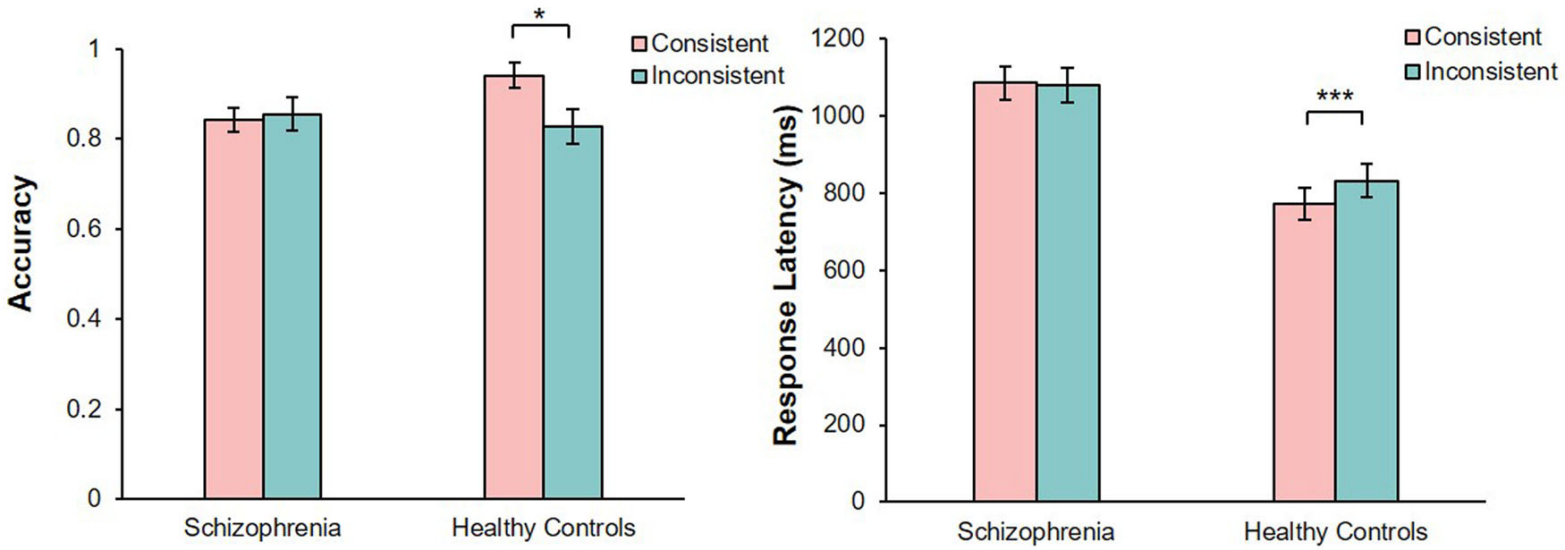
Shows the comparison of accuracy (left) and response latency (right) between consistent perspective and inconsistent perspective in patients with schizophrenia and healthy controls under the uncued self-avatar task. The error bars represent 1 standard error. *P < 0.05, ***P < 0.001.

A significant main effect of consistency was observed, *F* (1, 60) = 4.06, *P* < 0.05, partial *η*^2^ = 0.06. There were no significant main effects of group, *F* (1, 60) = 0.83, *P* = 0.37. There was a significant interaction between group and consistency, *F* (1, 60) = 6.80, *P* < 0.05, partial *η*^2^ = 0.10. The simple effect analysis showed that, healthy controls showed a higher accuracy rate in consistent perspectives condition (*M* = 0.94, 95% CI [0.88, 1.00]) than inconsistent perspectives condition (*M* = 0.83, 95% CI [0.75, 0.90], *t* (29) = 2.40, *P* < 0.05, Cohen’s *d*_*z*_ = 0.44. Patients with SZ showed no significant difference in accuracy rates between consistent perspectives (*M* = 0.86, 95% CI [0.78, 0.93]) and inconsistent perspectives (*M* = 0.84, 95% CI [0.79, 0.90], *t* (31) = 0.85, *P* = 0.40) (Fig. 3).

#### Response latency

There were significant main effects of group, *F* (1, 48) = 19.34, *P* < 0.001, partial *η*^2^ = 0.32 and consistency, *F* (1, 48) = 7.34, *P* < 0.01, partial *η*^2^ = 0.13. There was a significant interaction between group and consistency, *F* (1, 48) = 10.35, *P* < 0.001, partial *η*^2^ = 0.18. The simple effect analysis showed that, healthy controls showed longer response latency in the inconsistent perspectives condition (*M* = 831.82, 95% CI [745.51, 918.12]) than consistent perspectives condition (*M* = 772.22, 95% CI [689.71, 918.12]), *t* (25) = 6.52, *P* < 0.001, Cohen’s *d*_*z*_ = 1.29). Patients with SZ showed no significant difference in response latency between consistent perspectives (*M* = 1085.53, 95% CI [999.65, 1171.40]) and inconsistent perspectives (*M* = 1080.42, 95% CI [990.59, 1170.25], *t* (23) = 0.28, *P* = 0.78) (Fig. 3).

### Discussion

To examine the spontaneous process of visual perspective-taking in patients with SZ in Experiment 1b, we defined the identity of the avatar as the participants themselves, thereby emphasizing the salience of the perspective. The results showed that compared with the self-avatar’s inconsistent perspective, the accuracy and response latency of the healthy controls were lower than the consistent perspective. The patients’ response latency and accuracy did not differ between the consistent and inconsistent perspectives. We observed that, similar to healthy controls, patients with SZ did not display altercentric intrusions. This may indicate patients’ inability to adopt a perspective spontaneously regardless of whether the avatar represents the self or a stranger. However, according to O’Grady et al.^17^, self-identity does not draw patients’ attention to the avatar, which may have led to their lack of awareness of the potential relevance of perspective-taking. Therefore, we must consider the research findings of Simonsen et al.^12^ and enhance participants’ attention to perspective-taking using a cued dot perspective task.

## Experiment 2a: cued task of others’ perspective-taking in patients with schizophrenia

### Participants

An a priori power analysis was conducted using G*Power version 3.1.9.2^23^ to determine the minimum sample size required to test the study hypothesis. Results indicated the required sample size to achieve 80% power for detecting a medium effect (*f* = 0.2), at a significance criterion of *α* = 0.05, was *N* = 30 for 2 × 2 × 2 repeated measures in the analysis and interpretation of ANOVA. To avoid insufficient sample size due to participants abandoning the experiment, a total of 32 patients with SZ and 30 healthy controls were recruited for Experiments 2a and 2b. and 2b. All patients with SZ were recruited from the Tianshui Psychiatric Hospital, Gansu Province, China. The patients fulfilled the ICD-10 diagnostic criteria and were diagnosed by two experienced attending doctors. The exclusion criteria were substance abuse, history of severe brain trauma, and other physical diseases. All patients received antipsychotic medication (mean chlorpromazine equivalent = 261.65) during the experiment (Table 2). The recruitment and exclusion criteria for the participants were consistent with those in Experiment 1.

**Table 2:** Basic characteristics of participants.

	Schizophrenia (*n* = 33) (*M* ± SD)	Healthy controls (*n* = 35) (*M* ± SD)
Age (y)	30.06 ± 6.15	26.11 ± 10.95
Years of education	10.46 ± 3.58	12.75 ± 1.40
Gender (male/female)	16/17	20/15
Duration of illness (y)	7.00 ± 5.63	
SAPS	2.42 ± 5.76	
SANS	4.00 ± 5.53	

*SAPS* Scale for Assessment of Positive Symptoms, *SANS* Scale for Assessment of Negative Symptoms.

### Stimuli and design

We used the dot perspective task adapted from Samson et al.^12^ in this experiment. The stimulus pictures were the same as in Experiment 1. The experiment had a 2 (group: patients with SZ and healthy controls) × 2 (consistency: consistent and inconsistent) × 2 (perspectives: one’s own and other-avatar) factorial design.

### Cued visual perspective-taking task

**Fig. 4:**
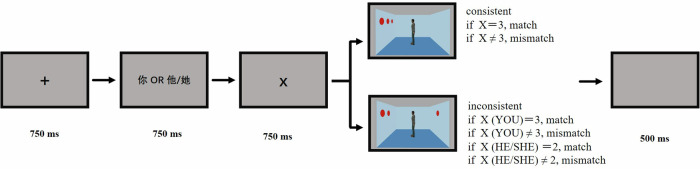
An illustration of the trial procedure in the cued other visual perspective-taking.

The participants were invited to the laboratory for the task, which was programmed using E-Prime 2.0. They were seated 60 cm from the monitor screen, with their head position fixed using a chin rest. Each trial began with the presentation of a black central fixation cross “+” for 750 ms, followed by the word “你” (Eng.: YOU) or “他/她” (Eng.: HE/SHE) for 750 ms, indicating which perspective had to be judged. Subsequently, one number from 0 to 3 was displayed for 750 ms, which cued the participant to verify the number of red discs in the next image. An image of the room was presented, and the trial continued until the participant pressed a key, which led to a 500 ms blank screen before proceeding to the next trial. The participant was required to press a key (F or J) to confirm whether the number of discs seen from the relevant perspective matched the previously seen number. Key presses were balanced among the participants (Fig. 4).

According to the experimental design, in the condition where the participant’s perspective matched the avatar’s, including one’s own and other-avatar perspectives, each perspective had 24 trials in which the answer was “yes” and 24 in which it was “no.” Similarly, when the participant’s perspective did not match the avatar’s perspective, there were 48 trials for each perspective. A total of 192 trials were presented for all the stimuli. To prevent the same conditions from occurring three consecutive times, all trials were presented pseudo-randomly during the experiment. In addition, 16 filler trials were included, in which the cue number was 0 and no red discs appeared in the image. Including the 12 practice trials, the total number of trials was 220. All the stimuli were presented across two blocks. Excluding the practice trials, each block contained 104 trials, and the participants took a break between the blocks. At the beginning of the practice phase, the assistant patiently explained the experimental content to the participants until they had fully understood it. The formal experiment took ~16–20 min.

In some trials in which the correct answer was “no,” the number of red discs did not correspond to either the participant’s perspective or the avatar’s, making these trials particularly easy to process and potentially confounding the results. Therefore, during the final analysis of results, we only analyzed conditions in which the correct answer was “yes.”

### Statistical analysis

We excluded the data from the practice trials. The response latency results were preprocessed, and trials with errors were removed. Only the response latencies falling within ± 3 standard deviations of each participant’s average response latency were analyzed. A total of 14 patients (10 with accuracy rates not exceeding 80% and 4 with average response latency exceeding 3000 ms) and 1 healthy control (with accuracy rates not exceeding 80%) were excluded from the analysis. All data were analyzed using IBM® SPSS® Statistics Version 23.0 via a 2 (group: patients with SZ and healthy controls) × 2 (consistency: consistent and inconsistent) × 2 (perspectives: one’s own and other-avatar) repeated measures ANOVA.

### Results

#### Accuracy rate

**Fig. 5:**
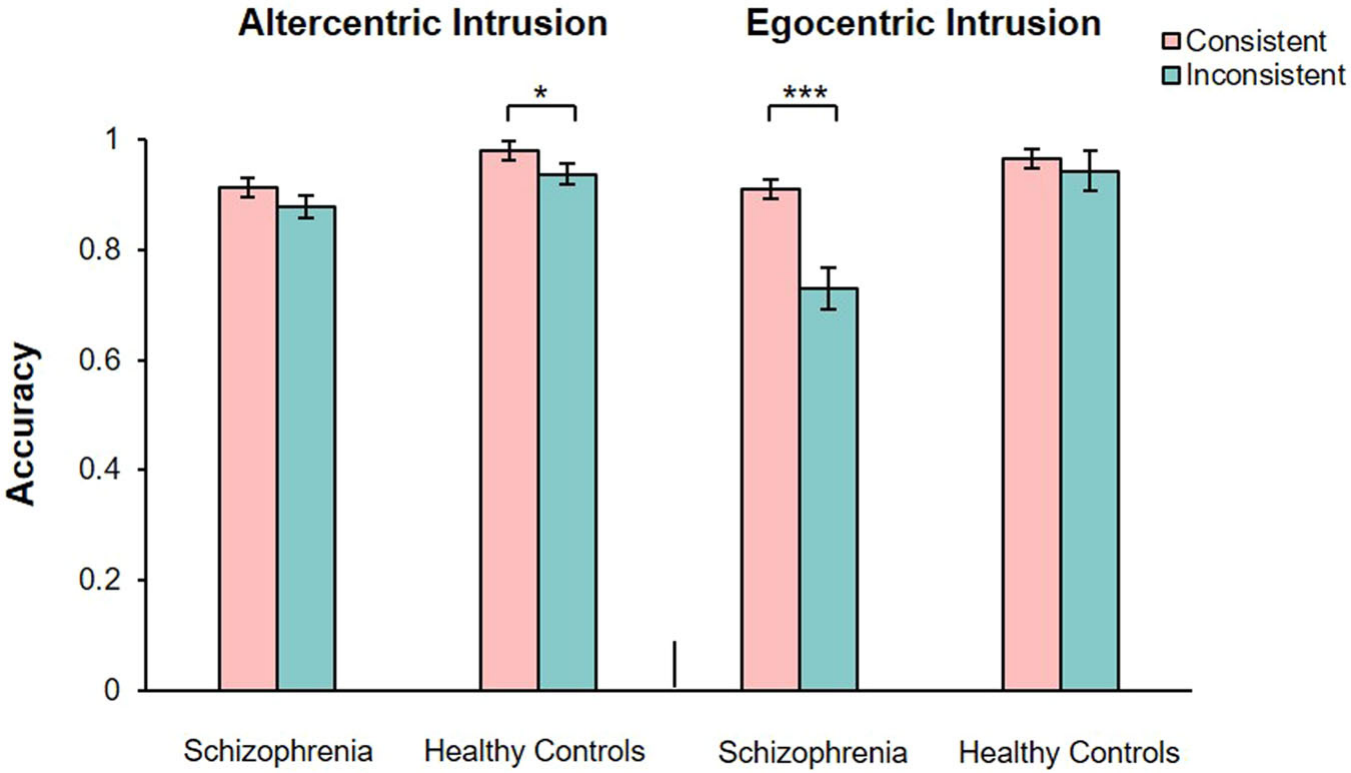
Shows the comparison of accuracy between consistent perspective and inconsistent perspective in patients with schizophrenia and healthy controls under the cued stranger avatar task. The error bars represent 1 standard error. *P < 0.05, ***P < 0.001.

A significant main effect of group, *F* (1, 66) = 18.24, *P* < 0.001, partial *η*^2^ = 0.22, consistency, *F* (1, 66) = 22.20, *P* < 0.001, partial *η*^2^ = 0.25, and perspectives was observed, *F* (1, 66) = 6.51, *P* < 0.05, partial *η*^2^ = 0.09. There was a significant interaction between group and perspectives, *F* (1, 66) = 5.22, *P* < 0.05, partial *η*^2^ = 0.07, group and consistency, *F* (1, 66) = 6.34, *P* < 0.05, partial *η*^2^ = 0.09, consistency and perspectives, *F* (1, 66) = 5.19, *P* < 0.05, partial *η*^2^ = 0.07. There was a significant interaction between group, consistency, and perspectives, *F* (1, 66) = 9.10, *P* < 0.01, partial *η*^2^ = 0.12. The simple effect analysis showed that, in the one’s own perspective condition, patients show no significant difference in accuracy rates between consistent perspectives (*M* = 0.91, 95% CI [0.88, 0.95]) and inconsistent perspectives (*M* = 0.88, 95% CI [0.84, 0.92], *t* (32) = 1.79, *P* = 0.08). Healthy controls showed higher accuracy rate in consistent perspectives condition (*M* = 0.98, 95% CI [0.95, 1.01]) than in the inconsistent perspectives condition (*M* = 0.94, 95% CI [0.90, 0.98], *t* (34) = 2.76, *P* < 0.01, Cohen’s *d*_*z*_ = 0.47. In the stranger avatar perspective condition, patients showed higher accuracy rate in consistent perspectives condition (*M* = 0.91, 95% CI [0.88, 0.95]) than inconsistent perspectives condition (*M* = 0.73, 95% CI [0.65, 0.81]), *t*(32) = 3.50, *P* < 0.01, Cohen’s *d*_*z*_ = 0.56. Healthy controls show no significant difference in accuracy rates between consistent perspectives (*M* = 0.97, 95% CI [0.93, 1.00]) and inconsistent perspectives (*M* = 0.94, 95% CI [0.87, 1.01], *t* (34) = 1.87, *P* = 0.07) (Fig. 5).

#### Response latency

**Fig. 6:**
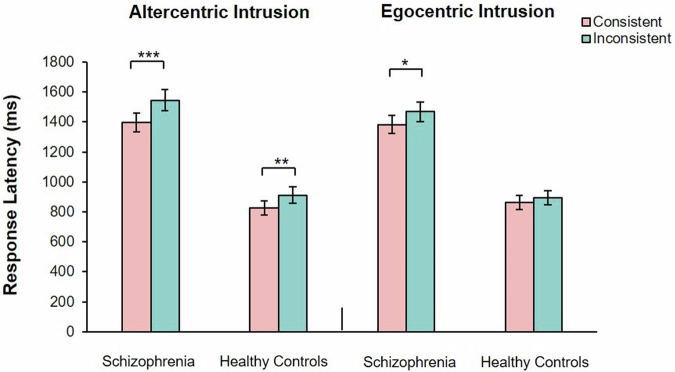
Shows the comparison of response latency between consistent perspective and inconsistent perspective in patients with schizophrenia and healthy controls under the cued stranger avatar task. The error bars represent 1 standard error. *P < 0.05, **P < 0.01, ***P < 0.001.

A significant main effect of group, *F* (1, 51) = 56.33, *P* < 0.001, partial *η*^2^ = 0.53, and consistency was observed, *F* (1, 51) = 25.47, *P* < 0.001, partial *η*^2^ = 0.33. There was no significant main effect of perspectives was observed, *F* (1, 51) = 0.83, *P* = 0.37. There was a significant interaction between group and perspectives, *F* (1, 51) = 25.47, *P* < 0.001, partial *η*^2^ = 0.33, group and consistency, *F* (1, 51) = 2.84, *P* = 0.10, consistency and perspectives, *F* (1, 51) = 7.20, *P* < 0.05, partial *η*^2^ = 0.12. There was no significant interaction between group, consistency and perspectives, *F* (1, 51) = 0.05, *P* = 0.82. The simple effect analysis showed that, in the one’s own perspective condition, all participants showed longer response latency in inconsistent perspectives condition (*M* = 1229.19, 95% CI [1137.97, 1320.47]) than consistent perspectives condition (*M* = 1112.11, 95% CI [1031.49, 1192.73], *t* (52) = 5.71, *P* < 0.001, Cohen’s *d*_*z*_ = 0.78. In the stranger avatar perspective condition, all participants showed longer response latency in inconsistent perspectives condition (*M* = 1181.57, 95% CI [1101.47, 1261.67] than consistent perspectives condition (*M* = 1122.35, 95% CI [1046.40, 1198.31], *t* (53) = 2.48, *P* < 0.05, Cohen’s *d*_*z*_ = 0.34) (Fig. 6).

### Discussion

We found that patients with SZ had a worse accuracy rate in inconsistent perspectives than in consistent perspectives for the other-avatar condition, which may indicate egocentric intrusion. Healthy controls did not show such a difference; however, in the condition for one’s own perspective, healthy controls demonstrated a worse accuracy rate in inconsistent perspectives than in consistent perspectives, which may indicate altercentric intrusion. In contrast, patients with SZ did not exhibit a difference between consistent and inconsistent perspectives. Patients with SZ had similar altercentric intrusion as healthy controls in their response latency index along with high egocentric intrusion. Experiment 2a indicated that the presence of a cued perspective can enable patients to become aware of the existence of perspectives and to spontaneously adopt the avatar’s perspective. However, inconsistencies in accuracy and response latency require further research to confirm the influence of the avatar. Experiment 2b examined the effect of the self-avatar on participants’ spontaneous perspective-taking by transforming the identity of the avatar into that of the participants themselves, thereby enhancing avatar salience.

## Experiment 2b: cued task of self-perspective-taking in patients with schizophrenia

### Methods

#### Stimuli, design, and procedure

**Fig. 7:**
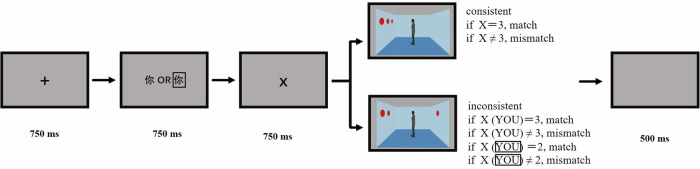
An illustration of the trial procedure in the cued self visual perspective-taking.

In this experiment, we used the same stimuli and procedures as in Experiment 2a, except for the avatar identity. The experimental instructions informed the participants that the avatar in the room represented themselves. To indicate the perspective, the word “你” (Eng.: YOU) or “你” appeared for 750 ms, indicating which perspective had to be judged. “你” represented the participant’s self-avatar in the room (Fig. 7). The study used a 2 (group: patients with SZ and healthy controls) × 2 (consistency: consistent and inconsistent) × 2 (perspective: one’s own and self-avatar) factorial design.

#### Statistical analysis

We excluded the data from the practice trials. The response latency results were preprocessed, and trials with errors were removed. Only the response latencies falling within ± 3 standard deviations of each participant’s average response latency were considered. In total, 20 patients (15 with accuracy rates not exceeding 80% and 5 with an average response latency exceeding 3000 ms) were excluded from the analysis. All data were analyzed using IBM® SPSS® Statistics Version 23.0 via a 2 (group: patients with SZ and healthy controls) × 2 (consistency: consistent and inconsistent) × 2 (perspectives: one’s own and self-avatar) repeated measures ANOVA.

### Results

#### Accuracy rate

**Fig. 8:**
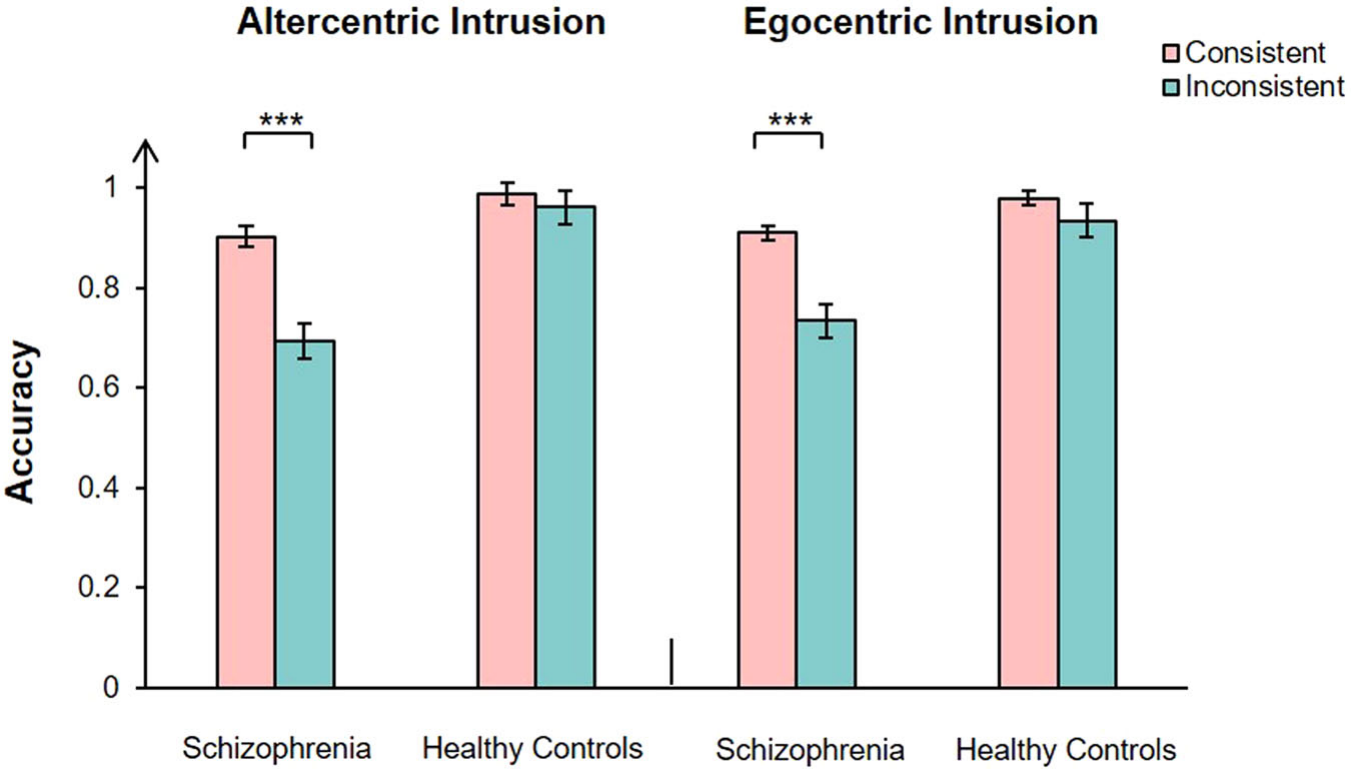
Shows the comparison of accuracy between consistent perspective and inconsistent perspective in patients with schizophrenia and healthy controls under the cued self-avatar task. The error bars represent 1 standard error. ***P < 0.001.

A significant main effect of group, *F* (1, 66) = 42.20, *P* < 0.001, partial *η*^2^ = 0.39, and consistency was observed, *F* (1, 66) = 46.84, *P* < 0.001, partial *η*^2^ = 0.42. There was no significant main effect of group perspectives was observed, *F* (1, 66) = 0.03, *P* = 0.87. There was a significant interaction between group and consistency, *F* (1, 66) = 22.45, *P* < 0.001, partial *η*^2^ = 0.25. There was no significant interaction between group and perspectives, *F* (1, 66) = 1.05, *P* = 0.31, consistency and perspectives, *F* (1, 66) = 0.03, *P* = 0.86. There was no significant interaction between group, consistency and perspectives, *F* (1, 66) = 0.59, *P* = 0.45. The simple effect analysis showed that, patients showed higher accuracy rate in consistent perspectives condition (*M* = 0.91, 95% CI [0.88, 0.95]) than inconsistent perspectives condition (*M* = 0.71, 95% CI [0.67, 0.76], *t* (65) = 5.74, *P* < 0.001, Cohen’s *d*_*z*_ = 0.71. Healthy controls show no significant difference in accuracy rates between consistent perspectives (*M* = 0.98, 95% CI [0.95, 1.02]) and inconsistent perspectives (*M* = 0.95, 95% CI [0.90, 1.00], *t* (69) = 2.28, *P* = 0.14)(Fig. 8).

#### Response latency

**Fig. 9:**
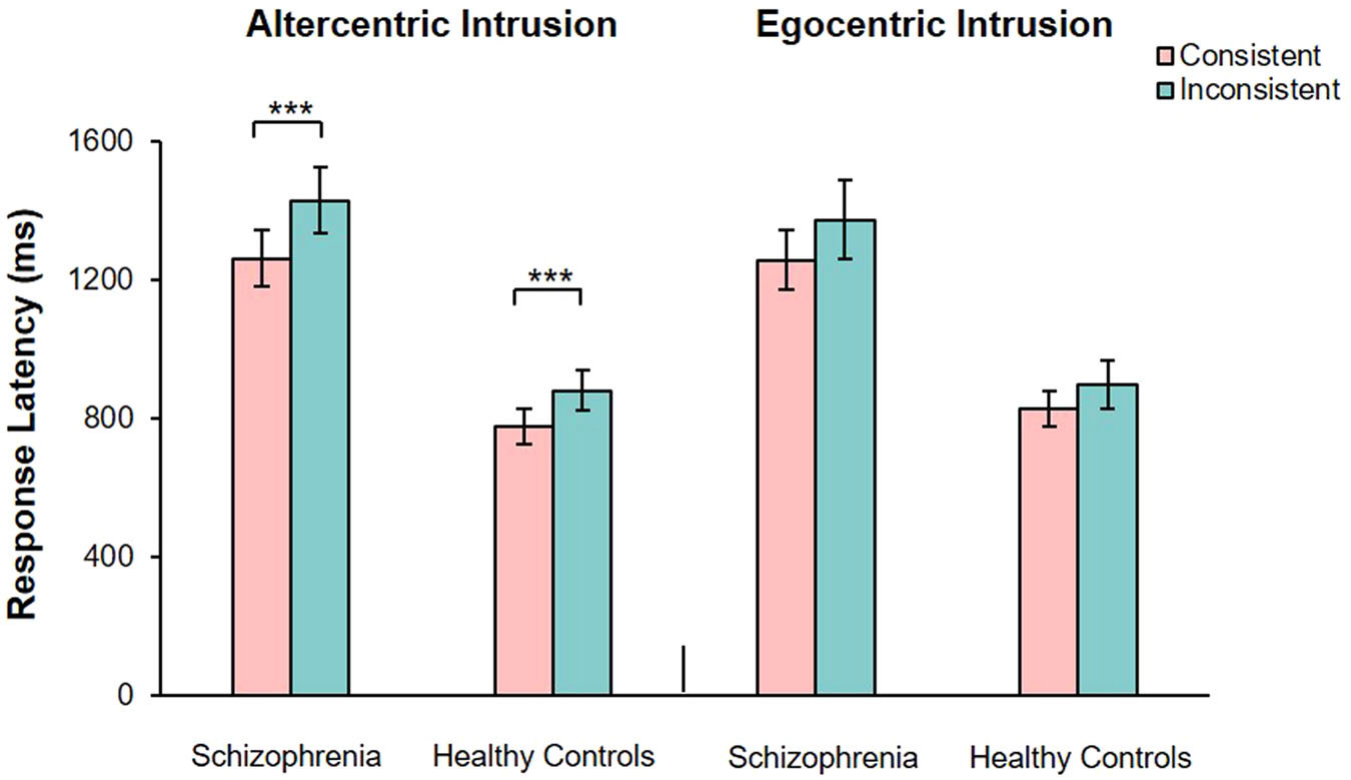
Shows the comparison of response latency between consistent perspective and inconsistent perspective in patients with schizophrenia and healthy controls under the cued self-avatar task. The error bars represent 1 standard error. ***P < 0.001.

A significant main effect of group, *F* (1, 46) = 21.00, *P* < 0.001, partial *η*^2^ = 0.31, and consistency was observed, *F* (1, 46) = 26.57, *P* < 0.001, partial *η*^2^ = 0.37. There was no significant main effect of perspectives was observed, *F* (1, 46) = 0.003, *P* = 0.96. There was no significant interaction between group and consistency, *F* (1, 46) = 1.60, *P* = 0.21, group and perspectives, *F* (1, 46) = 1.74, *P* = 0.19, consistency and perspectives, *F* (1, 46) = 1.89, *P* = 0.18. There was no significant interaction between group, consistency and perspectives, *F* (1, 46) = 0.06, *P* = 0.82. The simple effect analysis showed that, all participants showed longer response latency in inconsistent perspectives condition (*M* = 1144.85, 95% CI [1025.19, 1264.51]) than consistent perspectives condition (*M* = 1031.02, 95% CI [933.92, 1128.12], *t* (108) = 4.56, *P* < 0.001, Cohen’s *d*_*z*_ = 0.64) (Fig. 9).

### Discussion

Experiment 2b revealed that patients with SZ showed altercentric intrusion and had a higher accuracy rate and shorter response latency in consistent perspectives than in inconsistent perspectives of one’s own condition. In the self-avatar condition, patients with SZ showed egocentric intrusion and had a higher accuracy rate for consistent perspectives than for inconsistent ones. These findings suggest that avatar identity can influence the implicit visual perspective-taking abilities of patients with SZ. Specifically, increasing the salience of the avatar’s identity can enhance patients’ awareness of perspectives, thereby prompting them to actively adopt the other’s perspective. In addition, patients with SZ showed altercentric intrusion in accuracy rate and response latency in this experiment. This may indicate that patients have the ability to spontaneously adopt the avatar’s perspective.

### General discussion

The current study explored whether patients with SZ can spontaneously adopt others’ perspectives and the influence of avatars on this spontaneous process through an implicit visual perspective-taking task. We found that patients with SZ were capable of spontaneously adopting perspectives; however, their ability to do so for strangers was impaired, with their spontaneity being influenced by the identity of the individual whose perspective was being considered.

Visual perspective-taking is a fundamental component of ToM^24^, which humans gradually develop as they grow and mature. The findings of Experiment 1a indicating that patients with SZ were unable to spontaneously adopt the perspective of other avatars, it consistent with those of Kronbichler et al.^4^. In this experiment, participants were presented with only one avatar in the stimulus images and informed that the avatar was a stranger. In addition, the experimental instructions did not provide any details about perspective-taking. Healthy controls succeeded in spontaneously perceiving others’ perspectives, pointing toward an impairment in the spontaneous adoption of others’ perspectives in patients with SZ.

The following question remains: Is patients’ inability to spontaneously adopt others’ perspectives due to a disregard for other people or a lack of awareness of the existence of others’ perspectives? For patients with SZ, their illness affects most aspects of their cognitive abilities, such as their inability to focus well on objects in environment^21^. Consequently, they may fail to recognize the presence of additional information while performing experimental tasks, especially when such information seems unrelated to the current task. This may not reflect their inability to spontaneously adopt others’ perspectives but that they overlook doing so. According to O’Grady et al.^20^ in uncued tasks where the potential relevance of perspective-taking is unrecognized, spontaneous perspective-taking does not occur; however, when attention is drawn to the avatar, it does. Therefore, we conducted Experiment 1b.

To increase participants’ awareness of the avatar’s perspective in Experiment 1b, we changed the identity of the avatar from a stranger to the participants themselves. The existence of impairments in recognizing others’ perspectives has been verified in patients with SZ. Recent research has revealed that during the process of reflecting on others, patients with SZ exhibited reduced activation in the right temporoparietal junction—a brain region associated with self/other differentiation and social cognition. This indicated a failure in processing self- and other related information, which may lead to altered judgments of others’ behaviors^25^. This reduced activation could be a contributing factor to the impaired ability of patients with SZ to adopt others’ perspectives. However, Experiment 1b revealed that even when the avatar represented themselves, patients were unable to perceive the avatar’s perspective without cues, unlike healthy controls. These results indicated that patients with SZ were unable to adopt others’ perspectives spontaneously without being prompted. This may be due to impaired spontaneous perspective-taking or impairment in recognizing others. Another possibility is that the current task did not explicitly make the patients aware of the existence of perspectives. When patients perceive perspectives as irrelevant to the task at hand, they may not actively consider those of others^20^. Therefore, we decided to use the cued task to explore the participants’ ability to spontaneously adopt others’ perspectives.

Experiment 2a found that patients with SZ exhibited altercentric intrusion in response latency but not in accuracy rate. However, healthy controls showed altercentric intrusions in both indices. This may underscore patients’ difficulty in perceiving others’ perspectives spontaneously, which is consistent with the findings of Drayton et al.^19^ and Kronbichler et al.^4^. The response latency results indicated that patients with SZ may be influenced by others’ perspectives, albeit not as strongly as healthy controls. Simultaneously, it may also indicate that patients possess the ability to spontaneously adopt others’ perspectives. We conducted a final experiment to ascertain whether patients’ spontaneous adoption of others’ perspectives is influenced by avatar identity or spontaneous ability. The results revealed altercentric intrusion in the patients, as they spontaneously perceived a self-avatar perspective when the task required them to adopt their own perspective. Therefore, patients with SZ were capable of spontaneous perspective-taking.

Based on the combined results of all experiments, spontaneous perspective-taking was influenced by two factors in patients with SZ. The first factor was the awareness of perspectives. Previous studies have explicitly suggested that even in healthy individuals, the adoption of others’ perspectives may not always occur without explicit cues^26,27^. In daily life, to acquire valuable resources, individuals tend to spontaneously attend to information relevant to themselves. However, patients with SZ have been proven to exhibit attentional impairments, such as difficulties with sustained attention and controlling selection^28,29^. Recent research has indicated that patients with SZ exhibit stronger and spatially broader visual information filtering around their attentional focus compared with healthy individuals. This increased filtering of visual information beyond the focus of attention may affect higher-level behavioral, emotional, and cognitive domains^30^. Therefore, attentional impairment may hinder the spontaneous adoption of alternative perspectives unless their existence is explicitly emphasized for the patients.

The second most influential factor was the identity of the avatar. Previous studies have confirmed that nonsocial cues, such as triangles, fail to elicit spontaneous perspective-taking processes in patients with SZ^18^. The present study indicated that human avatars possessing social cues can also affect the spontaneity of patients’ perspective-taking processes, contingent upon the avatar’s identity. Keromnes et al.^31^ found that during visual recognition tasks, patients tended to focus on their own images, which led to significantly early self-recognition but delayed recognition of others. In visual perspective-taking tasks, patients’ difficulty in recognizing others affected their spontaneous perception of others’ perspectives. Compared with other avatars, embodying themselves was more likely to elicit their attention from the avatar’s perspective.

Our study had some limitations. First, in both the explicit and implicit visual perspective-taking tasks, the premise behind patients with SZ adopting others’ perspectives is that they understand that avatars in different locations may be able to see content that they cannot. We found that patients exhibited altercentric intrusion for the self-avatar identity, possibly indicating that they could comprehend that the view differed in different positions. However, this could also stem from patients’ abnormal self-perceptions^32^, as their arbitrary switching between the self and self-avatar may have influenced the experimental results. Therefore, future research must clarify patients’ ability to comprehend the differences between others’ and their own perspectives from diverse locations by incorporating cognitive neuroscience techniques to enhance the reliability of the findings. Second, a unique connection exists between individuals at high risk for SZ and patients with SZ, along with the abnormalities manifested in ToM^33^. Future research should delve into the performance of high-risk individuals in this task, particularly the uncued perspective-taking task, which may potentially serve as a method for the early identification of schizophrenic tendencies.

In summary, we found that patients with SZ exhibit a certain degree of altercentric tendency in implicit visual perspective-taking tasks, displaying altercentric intrusion when patients become aware of their own perspective existing in another spatial location within the scene. We suggest that patients with SZ are capable of spontaneously adopting perspectives, but this process is influenced by the degree of attention paid to the perspective and identity of others. This study provides insights into the social cognitive processes of patients with SZ. Future research should comprehensively look into the role of other individuals’ identities in the process of social cognition.

## Data Availability

The data presented in this study are available on request from the corresponding author.
